# Effects of Different Colored LEDs on the Enhancement of Biologically Active Ingredients in Callus Cultures of *Gynura procumbens* (Lour.) Merr.

**DOI:** 10.3390/molecules24234336

**Published:** 2019-11-27

**Authors:** Thang Tung Lian, Myat Myat Moe, Yong Ju Kim, Keuk Soo Bang

**Affiliations:** 1 Department of Lifestyle Medicine, College of Environmental and Bioresource Sciences, Jeonbuk National University, Iksan 54596, Republic of Korea; ttltungno90@gmail.com (T.T.L.); nationface@jbnu.ac.kr (Y.J.K.); 2 No. 35, Science St., Department of Botany, Dagon University, Dagon Myothit Township (East), Yangon 11451, Myanmar; dr.myatmyatmoe123@gmail.com; 3 Department of Oriental Medicine Resources, College of Environmental and Bioresource Sciences, Jeonbuk National University, Iksan 54596, Republic of Korea

**Keywords:** LEDs, elicitor, *Gynura procumbens*, callus, HPLC, LC-MS, cyanidin-monoglucosides

## Abstract

Conventional fluorescent lamps that are used in tissue culture are costly light sources, exhibiting excessive wavelength emission-bandwidth that must be replaced by alternative, less costly, and much lower power-consuming energy sources. The use of Light-Emitting Diodes (LEDs) is the best option due to their potential role as elicitors of secondary metabolite production in many plant models. *Gynura procumbens (G. procumbens)* is widely used for treating various diseases. Here, leaf explants were cultivated in MS medium that was supplemented with 0.5 mg/L of naphthaleneacetic acid (NAA) and 2.0 mg/L of benzylaminopurine (BAP) for 30 days under white, blue, and red LEDs. Secondary metabolites were analyzed by High Performance Liquid Chromatography (HPLC) and Liquid Chromatography-Mass Spectrometry (LC-MS). Blue LEDs elicited the highest antioxidant activity, total flavonoid, and phenolic content. Furthermore, the content of cyanidin-monoglucosides significantly increased under blue light.

## 1. Introduction

Light is one of the most crucial environmental factors that affect the developing plant and regulate its behavior [1]. Generally, fluorescent lamps, high-pressure sodium lamps, and metal halide lamps are used as the light sources for in vitro culture. However, they contain unnecessary radiation wavelengths that lead to low quality radiation for the stimulation of growth and they are reportedly responsible for as much as 65% of the total electricity consumed in tissue culture laboratories [2,3,4]. Recently, LEDs light has been widely used in agriculture as an alternative light source for plant growth and photosynthesis, as they conveniently show specific wavelength and bandwidth, long-life, and minimum heating, in addition to their small mass and volume [5,6]. Research has shown that it is mostly white, red, and blue light wavelengths that increase signal transduction and betalain biosynthesis [7]. It is estimated that almost 90% of plant development and physiology is influenced by the absorption of blue and red light (LEDs) [8,9]. In tissue culture studies, LED colors (wavelengths) or color combinations (wavelength combinations) that are commonly used include white, red, blue, and blue-red mixtures. Blue light plays a major role in chlorophyll biosynthesis, photosynthesis, stomatal opening, and the maturation of chloroplasts [10]. LEDs are preferred in in vitro growing environments, due to their durability, small size, low heat emission, and energy consumption; all of which make them ideal for in vitro plant propagation work [11]. Generally, anthocyanins are induced by visible, ultraviolet light and various types of LEDs lights [12,13]. Anthocyanins are phenolic molecules that cater natural colors to fruits and vegetables [14] and they are influenced by pH, temperature, and light [15]. A major role of phenolic compounds in preventing various chronic diseases is due to their properties as antioxidant, anti-carcinogenic, and anti-inflammatory compounds that have attracted the attention of many researchers for their health benefits, especially the antioxidant activity [16]. Anthocyanins have nutraceutical potential and they are used as active pharmaceutical ingredients; indeed, some of them are used as ancient practice for the treatment of several diseases. As a nutraceutical, the bioavailability of anthocyanin is crucial in maintaining good health and preventing disease [17]. The major pigment in different types of berry species is cyanidin, which has a natural reddish-purple (magenta) pigment, as in red sweet potato and in purple corn [18,19]. The application of high light intensity or hormonal combinations induce morphological changes, such as purple [20] or pink [21] calluses in in vitro callus culture.

*Gynura procumbens* (Lour.) Merr. (Asteraceae) is a well-known traditional medicinal plant in southeast Asia, whose leaves are succulent, elliptic and glossy purplish; it is approximately 10–25 cm high [22]. The leaves have served for food for more than many years, being generally served raw as salad. *G. procumbens* is widely applied for the treatment of inflammation, high cholesterol levels, high blood pressure, diabetes, kidney discomfort, and cancer [22]. This plant is especially well-known for its antioxidant activity [23,24,25] and its antihyperglycemic and antihyperlipidemic properties [26]. The advantages of using *G. procumbens* in the traditional manner have been supported by the identification and isolation of various medicinally important chemical constituents, including phenolic compounds, polyphenols, flavonoids, saponins, tannins, terpenoids, and essential oils [27,28]. Adding elicitors to in vitro plant cell, tissue, and organ cultures is a common practice that increases the production or induction of *de novo* synthesis of secondary metabolites, as, for example, anthocyanins, which are water-soluble pigments that are found in most plants [29,30].

To date, anthocyanin has not been reported in *Gynura procumbens*. As LED technology offers a possibility to enhance various compounds while using different wavelengths, we interestingly observed the presence of anthocyanin in calli extracts. Hence, the experiment continued to explore the induction of higher anthocyanin content under different wavelengths. This is the first report regarding the effects of LEDs on the production of anthocyanin.

## 2. Results and Discussion

### 2.1. Callus Induction

Optimum callus induction was observed after three weeks of culture on Murashige Skoog (MS) basal medium containing a combination of 0.5 mg/L of naphthaleneacetic acid (NAA) and 2.0 mg/L of benzylaminopurine (BAP) (Supplementary Table S1). This condition showed that the highest antioxidant activity (57.90 ± 2.32), total flavonoid content (TFC, 0.29 ± 0.14 mg/g), and total phenol content (TPC, 0.97 ± 0.03 mg/g) were observed in the MS basal medium that was supplemented with the same combination, at a statistically significant level (Supplementary Figure S1). In subsequent experiments, the same conditions that yielded optimum callus induction were used to induce the accumulation of anthocyanin from calli while using LEDs of different wavelength.

**Figure 1:**
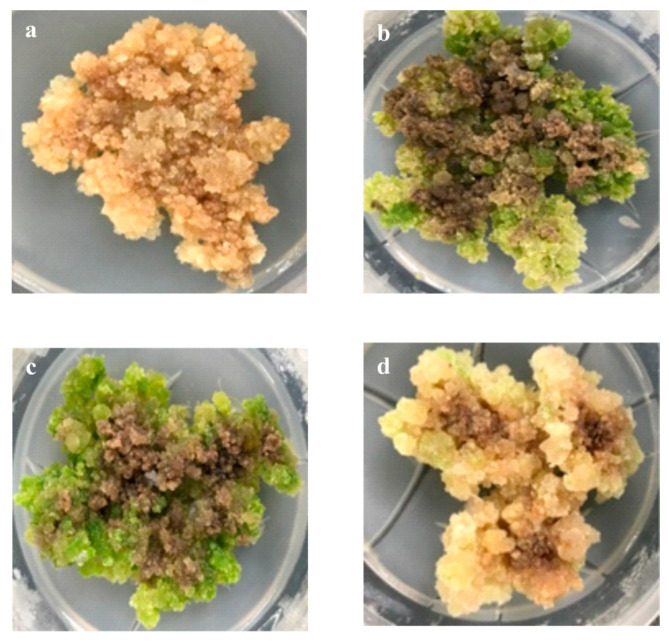
*Gynura procumbens* callus after 30-day culture under different Light-Emitting Diode (LED) lights. (**a**) Dark, (**b**) Blue, (**c**) White, and (**d**) Red.

The cultivation of callus under different colors (wavelengths) caused the morphological transformation of callus into brown, dark-green, and pinkish-brown by applying dark, blue, white, and red LEDs, respectively (Figure 1). The effect of different light spectra on the phenotype of calli grown under darkness appeared as paler or brown instead of green or red, when compared to calli that was grown under light. Overall, visual inspection can reveal the effect of LED wavelength, as calli did not show any organ development.

### 2.2. Effects of LED Lights on Antioxidant Activity, TFC, TPC, and Total Anthocyanin Content of G. Procumbens Calli

**Figure 2:**
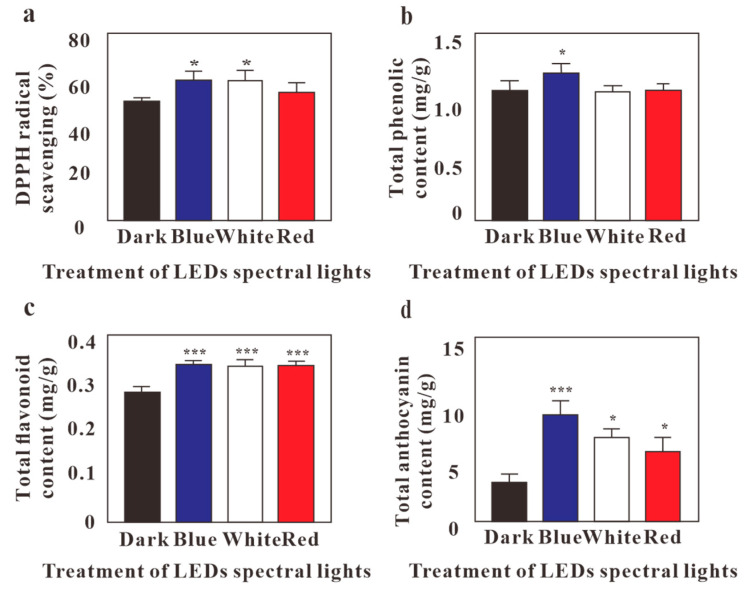
Effect of LEDs on callus culture for (**a**) DPPH free-radical savaging activity, (**b**) Total phenolic content, (**c**)Total flavonoid content, and (**d**) Total anthocyanin content of *G. procumbens* in callus culture. Values are means ± standard deviation (SD) from three replicates (*n* = 3). Ordinary one-way ANOVA followed by Dunnett’s multiple comparisons test were performed, where *p* < 0.05 and *p* < 0.001 are represented as * and ***, respectively.

LED effects on radical scavenging, as determined by the DPPH assay, are shown in Figure 2a and supplementary Table S2. When comparing the various LED lights tested, *G. procumbens* calli cultured under blue LEDs showed the highest radical-scavenging activity (*p* < 0.05) (Figure 2a). Thus, the expression of radical-scavenging activity can be sequenced as Blue > White > Red > Darkness (Supplementary Table S1). The development of antioxidant activity in calli grown under blue LEDs as compared to calli grown in darkness might be attributed to morphological differences between them, as the calli grown under blue LED light developed a dark-green color, while the dark induced gray calli (Figure 1). Phytochemical compounds may be present in plant extracts, which are capable of donating hydrogen ions to a free radical scavenger [31]. The maximum content of phenolic compound was observed in the *G. procumbens* callus that was grown under blue light (*p* <0.05), followed by cultures grown under red light, white light, and darkness (Figure 2b, Supplementary Table S2).

Flavonoid plant pigments consist of a group of secondary metabolites that have gained increasing attention due to their potential effects as helpful antioxidants for cancer and cardiovascular diseases, pathological disorders of gastric and duodenal ulcers, vascular fragility, allergies, and anti-viral and anti-bacterial activities [32]. Blue LEDs light showed the highest TFC (*p* < 0.001), followed by red (*p* < 0.001) and white (*p* < 0.001) LEDs (Figure 2c, Supplementary Table S2). In comparison, *G. procumbens* callus culture under dark conditions exhibited the lowest TFC among all of the culture extracts.

Anthocyanins are water-soluble glycosides and acylglycosides of anthocyanidins, a class of naturally occurring phenolic compounds. Naturally, 3-O-glycosides or 3,5-di-O-glycosides of cyanidin, delphinidin, peonidin, petunidin, pelargonidin, and malvidin are reportedly the most common anthocyanidins that are found in fruits and vegetables [33]. Anthocyanins have potential antioxidative, antiangiogenetic, anticancer, antidiabetic, antimicrobial, anti-obesity, and neuroprotective health benefits; additionally, they may help to prevent cardiovascular disease and improve visual health [34]. The results on the detection of total anthocyanin by a pH differential method showed that blue light was the most effective in increasing anthocyanin accumulation with (*p* <0.005) (Figure 2d, Supplementary Table S2). Therefore, *G. procumbens* calli demonstrated that blue light was the best option when compared to darkness, red, or white light (Figure 2).

### 2.3. HPLC and LC-MS Analysis on the Effects of LED Lights on Cyanidin-Monoglucosides Accumulation in Callus

**Figure 3:**
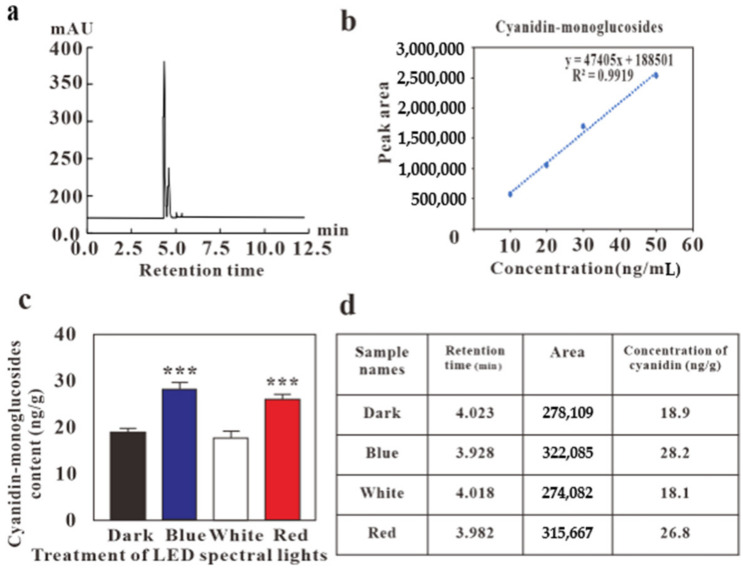
HPLC profile for the effect of LED lights for cyanidin-monoglucoside content in *G. procumbens* callus (**a**) peak of cyanidin-monoglucosides (**b**) standard curve (**c**) HPLC results (**d**) retention time and area of cyanidin-monoglucosides. Values are means ± standard deviation (SD) from three replicates (*n* = 3). Ordinary one-way ANOVA followed by Dunnett’s multiple comparisons test were performed, where *p* < 0.001 are represented as ***.

Cyanidin-monoglucoside is one of the most common anthocyanins found in plants. We found cyanidin-monoglucoside in the leaves of *G. procumbens* and determined its concentration by HPLC and LC-MS in calli. The extracts were analyzed by comparing the peak cyanidin-monoglucoside concentrations to the standard compound at 4.0 min., under dark at 4.023 min., under blue light at 3.928 min., under white light at 4.018 min., and under red light at 3.982 min. (Figure 3, Supplementary Figure S2). HPLC results showed that the extract from calli grown under blue light showed the highest accumulation of cyanidin-monoglucoside (28.2 ng/g), followed by calli cultured under red light, next by calli cultured under white light, and finally, the lowest content in cyanidin-monoglucoside was detected in calli kept in darkness (Figure 3c).

**Figure 4:**
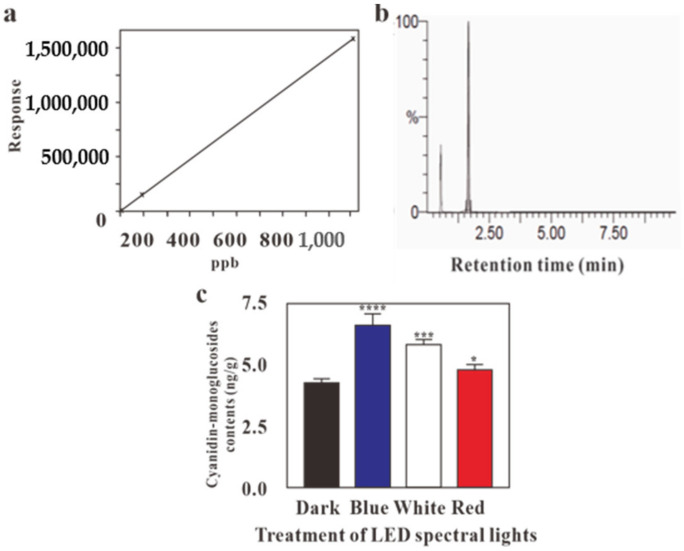
LC-MS profile for the effect of LED lights for cyanidin-monoglucoside content in *G. procumbens* calli. (**a**) Calibration curve of cyanidin-monoglucoside, (**b**) peak of cyanidin-monoglucosides, (**c**) effect of LEDs on cyanidin-monoglucoside content. Values are means ± standard deviation (SD) from three replicates (*n* = 3). Ordinary one-way ANOVA followed by Dunnett’s multiple comparisons test were performed, where *p* < 0.05, *p* < 0.001, *p* < 0.0001, are represented as *, *** and ****, respectively.

The cyanidin-monoglucoside concentration in *G. procumbens* calli cultured under blue light significantly increased while using LC-MS, as compared to the anthocyanin concentration that was observed under dark conditions. In contrast, red light had a relatively weak effect on anthocyanin production (Figure 4c). The composition of cyanidin-monoglucosides obtained using LC-MS under blue, red, and white light was 6.29, 4.80, and 5.98 ng/g, respectively, and significantly more strongly affected than calli grown under dark conditions (4.26 ng/g, *p* < 0.05). 

The application of LEDs in tissue culture ensures numerous advantages for the production of secondary metabolites, especially blue light, which proved to be the most effective in inducing anthocyanin accumulation when compared to red, white, or dark (Figure 3c). These data demonstrate that light, especially of specific wavelength, is an essential environmental factor for anthocyanin biosynthesis and might therefore be helpful for a faster and higher yield in industrial anthocyanin production. Further research still needs to be conducted on the effects of cross-talk or interaction among different light wavelengths on the biosynthetic pathway of anthocyanin in calli of *Gynura* species.

The HPLC and LC-MS analyses produced different results regarding the effects of red and white LED treatments. Anthocyanin production largely depends on pH, temperature, and light conditions. After the calli were freeze-dried at −60 °C, they were ground and the resulting powder was used to determine their anthocyanin content via HPLC analysis. For LC-MS analysis, fresh calli were homogenized and directly extracted. Despite the slight anthocyanin yield differences between the red and white LED treatments, the overall results showed that the blue LED treatment was the most effective in inducing anthocyanin production in *G. procumbens* calli; the HPLC and the LC-MS analyses both verified this. Moreover, fresh calli were more suitable for the in vitro induction of anthocyanin, as compared with freeze-dried calli. In their review article, Hasan et al. (2017) reported that red LEDs are more effective than blue LEDs in inducing anthocyanin production in apple skin that was grown in a greenhouse environmental control system. However, in other studies, the effects of blue LED radiation on enhancing the accumulation of anthocyanin have been observed in postharvest fruit, berries, and inflorescences [12,35,36,37,38,39]. Therefore, it can be suggested that the effect of LEDs on the production of anthocyanin depends on the plant species and variation of genetic expression, such as phytochrome (red light receptor) and cryptochrome (blue light receptor) [40].

## 3. Materials and Methods 

### 3.1. Plant Material

Wild *G. procumbens* plants were grown in pots (Plastic pots Planters; 17cm Diameter × 15cm × 12.3 cm Height). The stems were sterilized while using 15% NaOCl for 15 min. and then rinsed four times while using sterile distilled water. Sterile filter papers were used to absorb the remaining water, so that the stems were completely dry. Next, the stems were cut into 1.0 cm segments and planted on Murashige and Skoog (MS) solid basal medium in a Magenta box to obtain virus free plants. All the media were adjusted to pH 5.8 by adding NaOH and HCl and then autoclaved (DAIHAN Scientific) at 121 °C for 15 min. After autoclaving, the media were allowed to solidify on a clean bench until the experiment started. The nodal cultures were maintained in the culture room at 25 ± 2 °C.

### 3.2. Callus Establishment

After six weeks of culturing, the leaves were used as explants for callus induction. MS basal medium was supplemented with a combination of indolebutyric acid (IBA, 0.5, 1.0, 3.0, or 5.0 mg/L), naphthaleneacetic acid (NAA, 0.5, 1.0, 3.0, or 5.0 mg/L), 2.0 mg/L of Kinetin, and 2.0 mg/L of benzylaminopurine (BAP) for callus induction. The cultures were incubated under dark conditions for 30 days and observed twice a week under naked eye.

### 3.3. Spectral Light Treatments

Different LED (ODTech, Ltd., Korea) treatments were used as physical elicitors, including: red LEDs (24 h, 660 nm), blue LEDs (24 h, 460 nm), white LEDs (24 h, 400–700 nm), or darkness (24 h, as a control). Leaf calli were then transferred to a new medium, which included added NAA (0.5 mg/L) and BAP (2.0 mg/L). This was the optimum combination for callus induction. The calli were maintained in the culture room at 25 ± 2 °C under different spectral lights at 40 to 50 μMol m^−2^ s^−1^ photon flux measured with a Lux meter in the growth chamber (SU10, Jeiotech). After four weeks of culturing, the morphological differences among calli were observed under the naked eye, and they were harvested for subsequent extraction and analysis.

### 3.4. Sample Extraction

Induced calli were maintained and cultured on MS basal medium that was supplemented with a combination of 1.0 mg/L IBA and 2.0 mg/L Kin under different LED lights (red, blue, white) or darkness. After 30 days of culture, the calli were harvested, carefully separated from the medium, and then kept in a freeze-drier at −50 °C. Dried calli were ground and 1 g of each powder was soaked in 20 µL methanol 95% for 24 h before filtering through Whatman filter paper. The methanolic extracts were kept at −20 °C until analysis.

### 3.5. Analysis of Total Phenolic (TPC) and Total Flavonoid (TFC) Contents

One of the oldest methods for determining TPC in vegetables, fruits, and medicinal plants is Folin-Ciocalteu’s assay [41]. Briefly, 2% Na_2_CO_3_ and 50% of Folin-Ciocalteu reagent were prepared. Next, 100 µL of the sample and 2 mL of 2% Na_2_CO_3_ were mixed in a glass tube and left to stand for 3 min., after which, 100 µL of 50% FCR (Folin-Ciocalteu reagent, Sigma-Aldrich, St. Louis, MO, USA) was added to the tube and left standing for another 30 min. Absorbance was measured at 700 nm with a micro plate reader (SHIMADZU, UV-1601, Chiyoda-ku, Tokyo, Japan). The protocol of Fazal et al. [42] was followed with minor modifications to evaluate TFC. Briefly, 5% NaNO_2_, 10% of AlCl_3._6H_2_O, and 1N NaOH were separately prepared. Next, 250 µL of the sample was added to 1 mL distilled water and 75 µL of 5% NaNO_2_ and 15075 µL 10% of AlCl_3_· 6H_2_O were added and the mixture was left standing for 6 min. After the addition of 500 µL of 1N NaOH, the reaction was allowed to proceed for 11 min. and the absorbance was measured at 500 nm while using a micro plate reader. Gallic acid and catechin standard calibration curves were used for TPC and TFC estimation, respectively. The values were calculated as gallic acid (GE)/g and catechin (CAT)/g of dried weight (DW).

### 3.6. Determination of Antioxidant Activity

The measurement for free-radical scavenging potential was performed according to the method that was developed by Blois [43]. Briefly, 40 μL of extract mixed with 160 μL methanol were added to 1800 μL of DPPH solution and allowed to react for 30 min. in the dark. Absorbance was measured at 517 nm while using a micro plate reader. DPPH solvent was used as the control and methanol was used as a blank. The following equation was used to estimate free-radical scavenging activity:

% scavenging DPPH free radical = (AE/AD) *×*100% (1)

where AE represents absorbance at 517 nm and AD is the absorbance of DPPH solvent as control.

### 3.7. Analysis of Total Anthocyanin Content

The total anthocyanin contents of the calli were measured while using the pH-differential method [44]. For making 1.0 pH buffer (potassium chloride, 0.025 M), we measured the weight of 1.86 g KCL in a beaker and then added DW 980 mL. The pH was measured and adjusted to 1.0 with HCL. To adjust the pH buffer to 4.5 (sodium acetate, 0.4 M), we weighed 54.43 g of CH_3_CO_2_Na 3H_2_O in a beaker and combined it with 960 mL of distilled water. After the addition of 4.5 mL of pH = 1.0 or pH = 4.5 buffer solution to 0.5 mL of each sample (dilution factor, DF = 10) absorbance was measured at 510 and at 700 nm. Distilled water was used as a reference. Detailed information is provided in the supplementary materials (S1).

### 3.8. High Performance Liquid Chromatography (HPLC) Analysis

Calli that were grown under different LED spectral lights and in the dark were analyzed by HPLC while using a Shimadzu HPLC System (CBM-20A, LC-20A, SPD-20AD, and CTO-20A, Kyoto, Japan) with Nucleosil C18 reverse-phase (125-by-mm) column at 40 °C (Shimadzu). Detailed information is provided in the supplementary materials (S2).

### 3.9. Liquid Chromatography-Mass Spectrometry (LC-MS) Analysis

A XEVO-TQS#WAA250 triple quadrupole mass spectrometer (AB Sciex, Redwood City, CA, USA) with ESI source coupled with UHPLC (UltiMate 3000 RS, Thermo Fisher Scientific, Waltham, MA, USA) and a nitrogen generator (Parker, Lancaster, PA, USA) were used. Fresh calli that were grown under different LED lights or in darkness were harvested after 30 days and 1 g of each callus was extracted with 10 mL of 100% methanol. Detailed information is provided in the supplementary materials (S3).

### 3.10. Statistical Analysis

All of the experiments were repeated thrice. Graph Pad Prism (Windows, v7.0) was used for statistical analysis and making graphs. To define significant results, ordinary one-way ANOVA followed by Dunnett’s multiple comparisons test to separate means were performed (*p* < 0.05).

## 4. Conclusions

The optimum medium for callus induction was a combination of 0.5 mg/L NAA and 2.0 mg/L of BAP, as it also showed the highest antioxidant activity. The utilization of blue LED lights on in vitro callus cultures induced a high antioxidant activity and enhanced the accumulation of TPC, TFC, and TAC (Figure 2). The effects of different lighting conditions on in vitro cultures demonstrated that blue LEDs increased the anthocyanin accumulation. The system is cost-effective and low energy requiring; furthermore, it rendered a higher quantitative and qualitative yield when compared to the conventional method. Therefore, the utilization of LEDs satisfies the demand of various aspects in in vitro cultures involving commercial micropropagation, and it can be part of a reliable protocol for developing new drugs in addition to drug supplements.

## Supplementary Materials

Supplementary Materials are available online at https://www.mdpi.com/1420-3049/24/23/4336/s1.
