# Correction: Thang et al. Effects of Different Colored LEDs on the Enhancement of Biologically Active Ingredients in Callus Cultures of *Gynura procumbens* (Lour.) Merr. *Molecules* 2019, *24*, 4336

**DOI:** 10.3390/molecules28020884

**Published:** 2023-01-16

**Authors:** Thang Tung Lian, Myat Myat Moe, Yong Ju Kim, Keuk Soo Bang

**Affiliations:** 1 Department of Lifestyle Medicine, College of Environmental and Bioresource Sciences, Jeonbuk National University, Iksan 54596, Republic of Korea; 2 No. 35, Science St., Department of Botany, Dagon University, Dagon Myothit Township (East), Yangon 11451, Myanmar; 3 Department of Oriental Medicine Resources, College of Environmental and Bioresource Sciences, Jeonbuk National University, Iksan 54596, Republic of Korea

There were some errors in the original publication [1].

## Author Correction

It has changed and excluded the names and arrangements of authorship according to author contributions.

1. Se-Yeoun Cha is excluded from authorship.

2. Yong Ju Kim is excluded from Correspondence.

Original form:

Thang Tung Lian ^1,†^, Se-Yeoun Cha ^2,†^, Myat Myat Moe ^3^, Yong Ju Kim ^1,4,^* and Keuk Soo Bang ^1,4,^*

^1^ Department of Lifestyle Medicine, College of Environmental and Bioresource Sciences, Jeonbuk National University, Iksan 54596, Korea; ttltungno90@gmail.com

^2^ College of Veterinary Medicine and Center for Poultry Diseases Control, Jeonbuk National University, 79 Gobong-ro, Iksan 54596, Korea; seyeouncha@jbnu.ac.kr

^3^ No. 35, Science St., Department of Botany, Dagon University, Dagon Myothit Township (East), Yangon 11451, Myanmar; dr.myatmyatmoe123@gmail.com

^4^ Department of Oriental Medicine Resources, College of Environmental and Bioresource Sciences, Jeonbuk National University, Iksan 54596, Korea

* Correspondence: nationface@jbnu.ac.kr (Y.J.K.); ksbang@jbnu.ac.kr (K.S.B.); Tel.: +82-63-850-0745 (Y.J.K.); +82-63-850-0742 (K.S.B.); Fax: +82-63-850-0741 (K.S.B.)

† These authors contributed equally to this work.

Correct form:

Thang Tung Lian ^1^, Myat Myat Moe ^2^, Yong Ju Kim ^1,3^ and Keuk Soo Bang ^1,3,^*

^1^ Department of Lifestyle Medicine, College of Environmental and Bioresource Sciences, Jeonbuk National University, Iksan 54596, Republic of Korea

^2^ No. 35, Science St., Department of Botany, Dagon University, Dagon Myothit Township (East), Yangon 11451, Myanmar

^3^ Department of Oriental Medicine Resources, College of Environmental and Bioresource Sciences, Jeonbuk National University, Iksan 54596, Republic of Korea

* Correspondence: ksbang@jbnu.ac.kr; Tel.: +82-63-850-0742; Fax: +82-63-850-0741

**Author Contributions:** Formal analysis, T.T.L., K.S.B.; Investigation, T.T.L., K.S.B; Resources, T.T.L.; Validation, T.T.L, M.M.M., Y.J.K., K.S.B.; Writing - original draft, T.T.L, M.M.M., Y.J.K., K.S.B.

## Text Correction

1. Section 2.1. Callus Induction. The unit has been corrected (on the 2nd page on the 40th line) as follow: 0.29 ± 0.14 mg/g
2. Section 2.3. HPLC and LC-MS Analysis on the Effects of LED Lights on Cyanidin-Monoglucosides Accumulation in Callus. The values and unit have been corrected (on the 4th page on the 19th line) as follow: 28.2 ng/g
3. Section 3.4. Sample Extraction and Figure S1: Effect of various plant growth regulator combinations on callus culture for (**a**) DPPH free radical scavenging activity (**b**) TFC and (**c**) TPC. Kn (abbreviation of Kinetin) has been correct as Kin, not only in the main text, but also in the legend of this article for the unification.

## Error in Figure

1. Figure 3. HPLC profile for the effect of LED lights for cyanidin-monoglucoside content in *G. procumbens* callus. The chromatogram of (**a**) panel and the calibration curve of (**b**) panel have been changed, the unit (µg/g) of (**c**) panel has been corrected as ng/g, and the area of the column represented at (**d**) panel has been changed.

2. Figure 4. LC-MS profile for the effect of LED lights for cyanidin-monoglucoside content in *G. procumbens* calli. The unit (µg/g) of (**c**) panel has been corrected as ng/g, and the values of the Y axis have been changed. However, both the caption and the discussions regarding Figure 3 and Figure 4 have remained unchanged:

**Figure 3:**
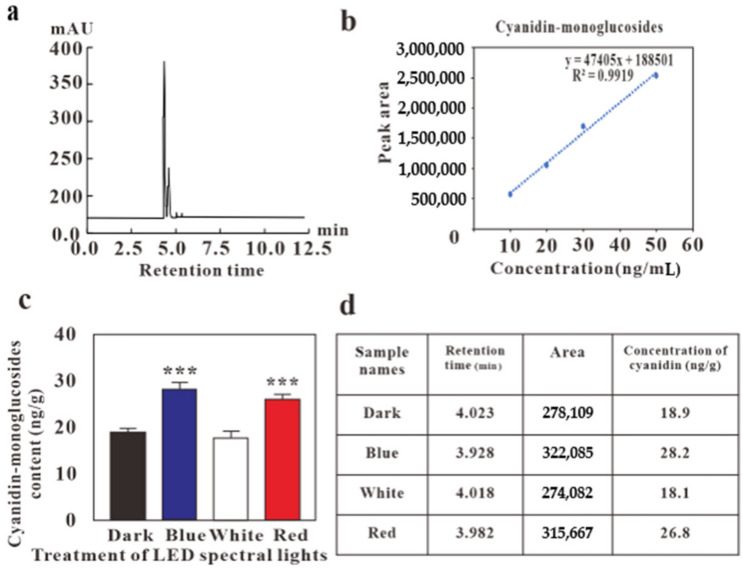
HPLC profile for the effect of LED lights for cyanidin-monoglucoside content in *G. procumbens* callus (**a**) peak of cyanidin-monoglucosides (**b**) standard curve (**c**) HPLC results (**d**) retention time and area of cyanidin-monoglucosides. Values are means ± standard deviation (SD) from three replicates (*n* = 3). Ordinary one-way ANOVA followed by Dunnett’s multiple comparisons test were performed, where *p* < 0.001, are represented as ***.

**Figure 4:**
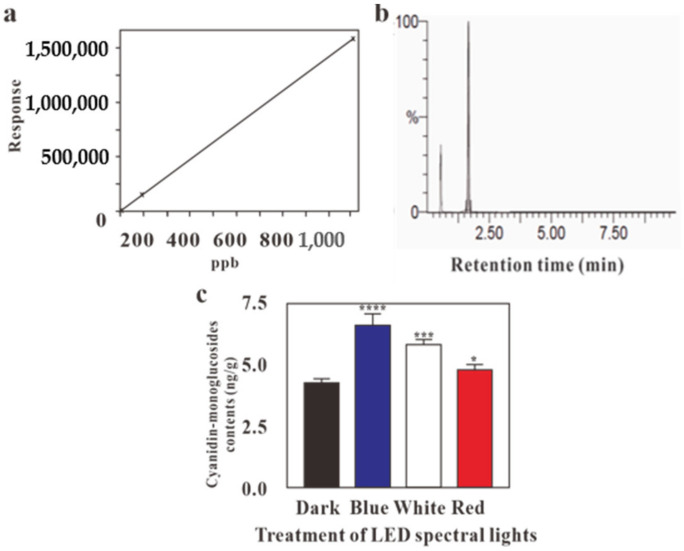
LC-MS profile for the effect of LED lights for cyanidin-monoglucoside content in *G. procumbens* calli. (**a**) Calibration curve of cyanidin-monoglucoside, (**b**) peak of cyanidin-monoglucosides, (**c**) effect of LEDs on cyanidin-monoglucoside content. Values are means ± standard deviation (SD) from three replicates (*n* = 3). Ordinary one-way ANOVA followed by Dunnett’s multiple comparisons test were performed, where *p* < 0.05, *p* < 0.001, *p* < 0.0001, are represented as *, *** and ****, respectively.

## The Supplement Has Also Been Corrected in Order to Prevent Confusion to Readers

1. S2. HPLC analysis

- The literature has been added (on the 1st line on the 1st page) as follow: HPLC analysis was used by modifying the method of Madhavi et al. [1].
- The text has been corrected (on the 5th line and on the 7th line on the 1st page) as follows: The 5th line: Solvents were 100% Acetonitrile (A) and 1% phosphoric acid (B). Separation was obtained by an isocratic elution at 0.7 mL/min (% ratio of A:B = 20:80). The 7th line: detected at 200 nm~400 nm.

2. Table S4. HPLC conditions for the analysis of cyanidin-monoglucosides in Gynura procumbens have been corrected as follow.

**Table:** 

Instrument	Shimadzu HPLC System (CBM-20A, LC-20A, SPD-20AD, and CTO-20A, Japan)
Column	C18
Column temperature	40 °C
Final concentration of mobile phase (isocratic)	A: acetonitrile 20% B: phosphoric acid 0.8% in water
Flow rate	0.7 mL/min
Injection volume	10 µl
UV wavelength	200 nm~400 nm
Run time	25 min

3. Table S5: The observation of the differences between the extraction of fresh and dried callus on the yield of cyanidin-monoglucosides, as well as Figure S2: HPLC profile standard curve of cyanidin-monoglucosides. Figure S4: The comparative peak area covered by freeze dried and fresh callus extract grown in LEDs is overlapped with the main figures, and which is unnecessary for this article, has therefore been deleted.

The authors apologize for any inconvenience caused to the readers by this change. The manuscript will be updated, with a reference to this correction and stating that the scientific conclusions are unaffected. This correction was approved by the Academic Editor. The original publication has also been updated.
